# From Paper to Digital Medical Documentation in the Field: The Rapid Development and Deployment of the Digital Casualty Card System During a War

**DOI:** 10.2196/79808

**Published:** 2026-07-24

**Authors:** Gur Hadar, Gil Shimon, Danielle Akler, Yochay Raviv, Irina Radomislensky, Zivan Aviad Beer, Avi Benov, Itay Ketko, Elon Glassberg, Ofer Almog

**Affiliations:** 1 Israel Defense Forces Medical Corps Ramat Gan Israel; 2 Gray Faculty of Medical and Health Sciences, Tel Aviv University Tel Aviv Israel; 3 The National Center for Trauma and Emergency Medicine Research The Gertner Institute for Epidemiology and Health Policy Research Sheba Medical Center Ramat Gan, Tel Aviv Israel; 4 Department of Military Medicine, Faculty of Medicine, Hebrew University Jerusalem Israel; 5 The Azrieli Faculty of Medicine, Bar-Ilan University Safed Israel; 6 Uniformed Services University of the Health Sciences Bethesda, MD United States; 7 Tel Aviv Sourasky Medical Center Tel Aviv Israel

**Keywords:** prehospital medical documentation, combat casualty care, digital documentation, prehospital trauma care, electronic health record

## Abstract

**Background:**

The accurate documentation of medical treatments for combat-injured personnel has historically posed significant challenges for prehospital medical teams. During recent US Army conflicts in Afghanistan and Iraq, only 18%-25% of casualties had any form of prehospital documentation. In the Israel Defense Forces (IDF), traditional manual and paper-based documentation has proven inefficient. During the 2014 Israel-Hamas conflict in Gaza, the completion rate for full documentation was notably low; only 11% (82/704) of casualties had casualty cards from the field. The sudden outbreak of the 2023-2024 Israel-Hamas war required an immediate re-evaluation of battlefield medical documentation practices. The IDF identified an urgent need for an innovative documentation approach to address the challenges of managing and tracking casualties in high-pressure scenarios. The integration of this system underscores the importance of real-time, robust documentation in improving continuity of care, minimizing medical error, and enhancing operational efficiency.

**Objective:**

This study outlines the rapid development and deployment of the Digital Casualty Card System (DCCS), designed to enhance the accuracy and efficiency of field documentation by medical teams during the 2023-2024 Israel-Hamas war.

**Methods:**

The DCCS was designed to streamline real-time medical data capture, enhance information transfer along the evacuation chain, and improve battlefield casualty care. A strategic decision was made to prioritize rapid deployment by focusing on a user-friendly, digital application, deliberately excluding advanced features such as sensor integration and real-time data transfer between echelons. This system became operational within 2 weeks of the project’s initiation and comprises military-grade tablets embedded with a dedicated software application for documenting casualty status and plastic memory cards worn around the casualty's neck for data transfer between medical teams. This study uses patient data from the IDF Trauma Registry, relying on data from point of injury casualty cards (DCCS), after-action reports, and data entry by on scene and en route providers.

**Implementation (Results):**

Overall, since the beginning of the distribution, over 700 DCCS kits were embedded in combat units, medical evacuation units, and training units. During the ground operation in Gaza, out of 2984 casualties, 1175 (39%) arrived with DCCS documentation.

**Conclusions:**

The rapid development and deployment of DCCS during the ongoing war proved to be feasible and contributed to the substantial improvements in both documentation rates and the quality of data collected in the field compared to traditional paper-based casualty cards.

## Introduction

Medical documentation has historically posed significant challenges for prehospital medical teams, particularly during high stress events such as under combat or mass casualty emergencies. Faced with the demands of treating severe, traumatic, time-sensitive injuries in the out-of-hospital setting, the prioritization of documentation of medical status and treatment has often been low, resulting in consistently low completion rates worldwide. During the US military conflict in Afghanistan, only 7% (24/363) of eligible casualties had completed Tactical Combat Casualty Care card documentation from 2013 to 2014 [1]. In other studies, only 18%-25% of casualties from conflicts in Afghanistan and Iraq had any form of prehospital documentation [2]. Similarly, Eastridge et al [3] reported that only 13% of US Army patients had their Tactical Combat Casualty Care card completed before advancing to the next medical echelon. A comparable trend was observed in the Israel Defense Forces (IDF) during the 2014 Operation in Gaza, with only 82 out of 704 (11%) casualties arriving at hospitals with prehospital medical documentation. Furthermore, most of the documentation was conducted by medical evacuation (MEDEVAC) units, with documentation rates at the point of injury being even lower. The result is an impairment in the continuity of care and level of medical care, which may be harmful for the casualties. For example, in several instances, casualties were administered a drug multiple times due to poor documentation and communication [4].

High-quality medical documentation on the battlefield is critical for several key reasons:

1. Ensuring continuity of care: accurate, timely information transfer along the evacuation chain is essential for optimal casualty care. Detailed documentation preserves continuity, ensuring each team knows prior interventions and current status. This enables informed decisions under challenging conditions and helps reduce preventable battlefield deaths. [5,6].
2. Conversion of data into knowledge: systematic collection of injury data from point of injury through all care stages enables comprehensive analysis and continuous improvement of prehospital medicine. It also yields operational insights on the protective measures and injury mechanisms, informs tactical decisions, and supports objective assessment of caregivers’ performance for targeted feedback and development.
3. Reduction of evacuation time and enhancing the efficiency of information transfer: an efficient documentation system streamlines casualty evacuation and minimizes delays between echelons of care. This improves both the speed and quality of treatment and reduces the time evacuation teams spend in combat zones, enhancing their safety and operational effectiveness.

The traditionally used paper casualty cards were prone to damage, loss, and misplacement, proving impractical due to their lack of durability and legibility in challenging tactical environments. Additionally, the use of separate forms at each echelon disrupted data continuity and limited information transfer between levels of care.

Many civilian and military organizations have tried to develop digital medical documentation systems, but few have achieved widespread operational use. The main barriers are organizational and technological: integrating sensors and syncing data with monitors in austere, secure settings is difficult, and fully embedding new tools into prehospital and combat workflows and culture, particularly among medical teams who may initially perceive them as cumbersome or disruptive, is even harder. For example, the United States Air Force Research Laboratory developed the Battlefield Assisted Trauma Distributed Observation Kit to improve documentation at the point of injury and during en route care, illustrating ongoing organizational efforts to address these challenges [7].

The IDF Medical Corps (IDF-MC) seeks to reduce preventable deaths by strengthening prehospital trauma care. The sudden outbreak of the Israel-Hamas war on October 7, 2023, with rapid mobilization of combat units and intense fighting exposed critical gaps in battlefield documentation and demanded immediate change. Drawing on prior conflicts, the IDF-MC rapidly designed and fielded the Digital Casualty Card System (DCCS) to improve the accuracy, availability, and efficiency of field documentation. The system highlights how robust, near real-time documentation can support continuity of care, reduce errors, and enhance operational performance in both mass-casualty events and routine operations. We believe that sharing our experience in the rapid development and deployment of DCCS may help other militaries and organizations prepare for and manage large-scale emergencies more effectively.

## Methods

### Overview

In the 5 years preceding the war, significant efforts were made to develop a digital casualty documentation system. This initiative sought to establish a network-based solution that integrated medical monitoring, data processing, and communication capabilities to facilitate information transfer throughout the evacuation chain and to support informed decision-making [8]. However, this system had not reached operational maturity as the war commenced. Consequently, there was an urgent need for a practical and immediate solution to replace the traditional paper casualty cards. To meet this need, the development of DCCS was guided by several key considerations to address the urgent operational demands.

A Minimal Viable Product approach was adopted, recognizing the solution’s ad hoc nature and limitations. In collaboration with civilian volunteers from an Israeli health care software company (K Health), a dedicated IDF-MC team developed DCCS to improve the accuracy and efficiency of field documentation. DCCS was built to streamline real-time data capture, enhance information transfer along the evacuation chain, and improve battlefield casualty care. To enable rapid deployment, the team focused on a simple, user-friendly application and deliberately excluded advanced features such as sensor integration and real-time data exchange between echelons. This Minimal Viable Product approach demonstrated that, with clear priorities, rapid feedback and tight collaboration between end users and developers, a functional field-ready product could be delivered within weeks, succeeding where more complex systems have often struggled.

### General Approach and Usability

The DCCS was designed as a simple, intuitive tool for documenting casualties in the field. Military-grade tablets run a dedicated application, which connect to a plastic near field communication (NFC) “dog tag” placed around the casualty’s neck by life support providers at the point of injury. The “dog tag” stores the digital record when direct tablet contact cannot be established. This allows medical teams at each echelon to record status and treatments, append follow-up information, and deliver a complete record to hospitals in Israel, supporting continuity and accuracy of care and data transfer.

**Figure 1:**
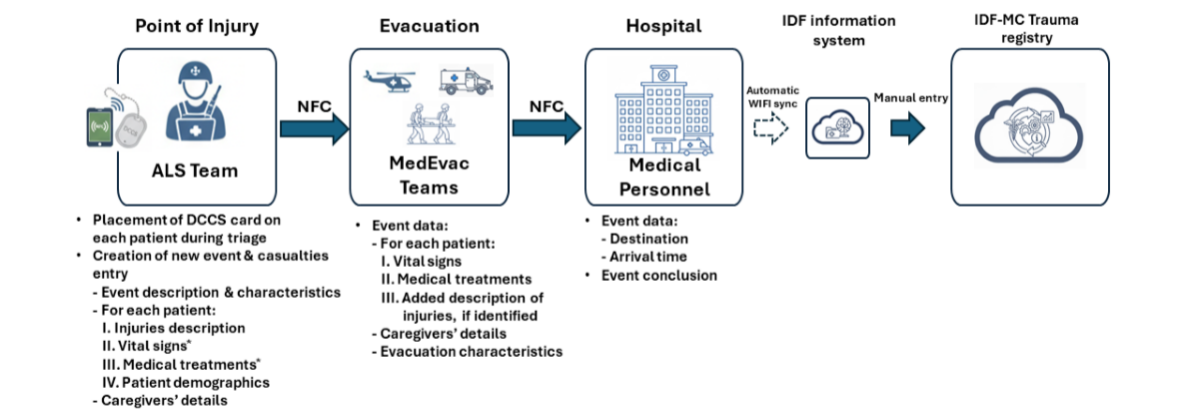
DCCS data flow through echelons of care during the 2023-24 Israel-Hamas war. ALS: advanced life support; DCCS: Digital Casualty Card System; IDF: Israel Defense Forces; IDF-MC: Israel Defense Forces Medical Corps; NFC: near field communication; *periodically.

Hospital data are then transferred to an information system that interfaces with the IDF-MC Trauma and Combat Medicine Branch and the IDF Trauma Registry (IDF-TR). Because of security, technical constraints, and the urgent rollout, hospital databases were not directly connected to the military network; so DCCS records had to be uploaded manually—a limitation expected to be resolved in future updates. Even without full integration, the DCCS already supports continuous learning, strengthens legal accountability through standardized records, reduces evacuation delays and data loss, and ensures that medical data travel with the casualty. Figure 1 presents a schematic overview of the DCCS workflow described above.

### Hardware

**Figure 2:**
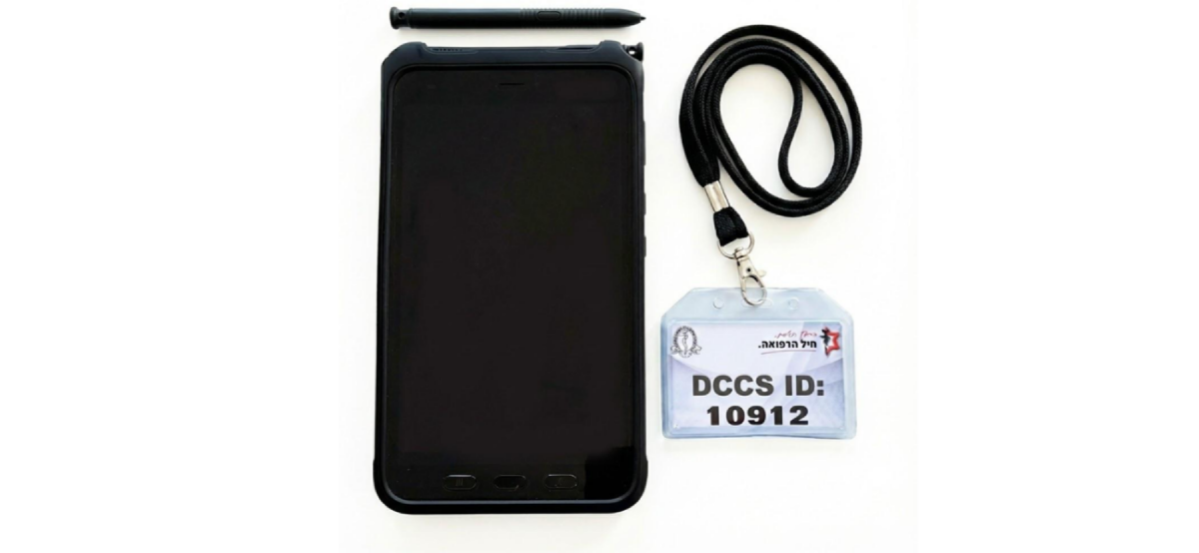
Digital Casualty Card System (DCCS) v3 tablet and near field communication dog tag.

The DCCS hardware consists of military-grade, Android-based tablets, and plastic NFC “dog tags.” Because battlefield communication is tightly restricted, only an air-gapped platform was permitted; so, communication-disabled tablets were used solely for medical documentation. Compact and lightweight, they are equipped with NFC to transfer data to and from the digital “dog tags,” which serve as portable casualty records. Any compatible tablet can access and update the tag, ensuring that “data moves with the casualty” and that data are continuously added at each echelon of care. Tablets were also preloaded with manuals, educational materials, operational lessons, and clinical guidelines, exploiting the fact that they remain with frontline caregivers throughout deployment. The tablet and digital dog tag are shown in Figure 2.

### Software

The DCCS software was designed to be user-friendly. The application requires medical teams to enter comprehensive casualty data with minimal manual actions. By analyzing our data registries during casualty treatment, we sought to achieve a balance between capturing all clinically important information and ensuring that the documentation process would be practical under operational pressure. This required repeated refinement of the interface, prioritizing usability while preserving the critical data needed for continuity of care and future performance improvement. The application supports the documentation of vital signs, treatments, and medical procedures administered to the casualty, along with patient identification, details of the medical team on the scene, and specifics of the injury, such as the mechanism, affected anatomical areas, and bodily systems involved.

**Figure 3:**
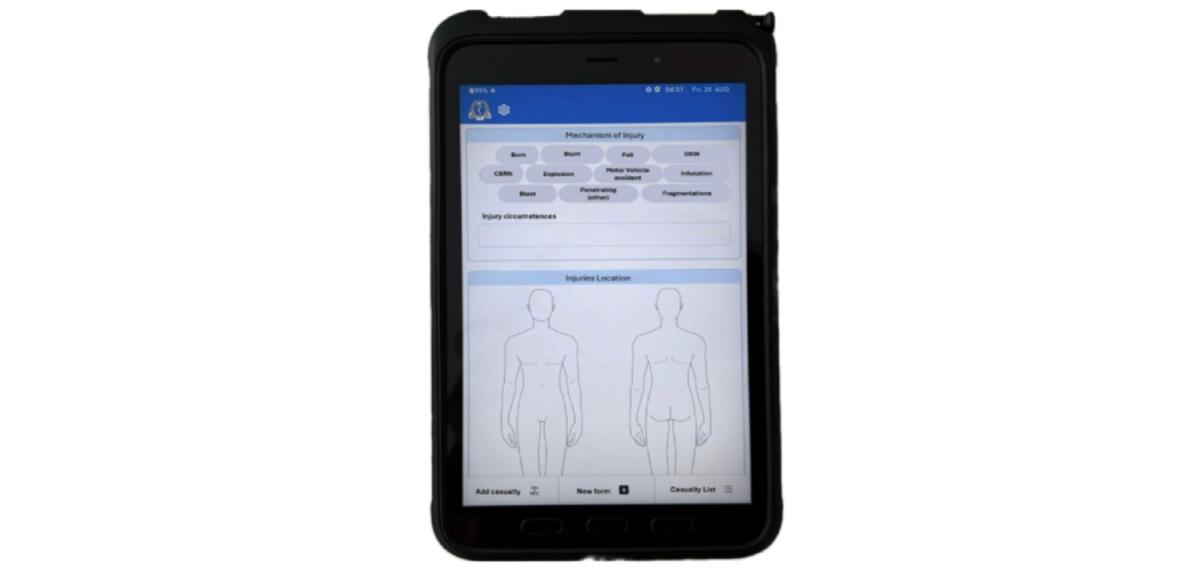
Digital Casualty Card System injury characteristics interface.

The manual data entry process was designed to be highly intuitive and touchscreen-based to enhance usability. For example, clinicians can indicate injured areas on a graphical representation of the human body and select options from predefined multiple-choice lists, rather than inputting text manually. This approach is intended to streamline and shorten the time of data entry, reduce errors, and improve the efficiency and accuracy of medical documentation. However, some data such as vital signs must still be entered manually as text. Figure 3 presents the user interface of DCCS.

### Implementation Process

Within 2 weeks of the project's initiation, the first version of the system entered the implementation process as a pilot with various MEDEVAC units, ground forces MEDEVAC units, and aeromedical evacuation unit. This pilot phase followed an agile methodology, allowing for iterative development and refinement based on feedback.

A designated instructor from the development team oversaw DCCS deployment. He visited frontline units and bases, provided on-site, hands-on training, and demonstrated practical use of the system. Each unit received structured instruction, and feedback from the field was relayed to Trauma and Combat Medicine Branch and developers for rapid refinement. Medical staff were encouraged to practice independently to gain proficiency under battlefield conditions. In parallel, dedicated training teams at the IDF Military Medicine Academy and other facilities integrated DCCS into formal curricula, addressing a long-standing gap in emphasizing documentation and reinforcing its clinical and operational importance. By embedding DCCS training within medical education, the initiative sought to instill a deeper understanding of the clinical and operational importance of proper documentation. Initially, 2 previous configurations of the DCCS were developed but subsequently upgraded due to negative feedback. The 3 different versions and their key features are described in Table 1.

**Figure 4:**
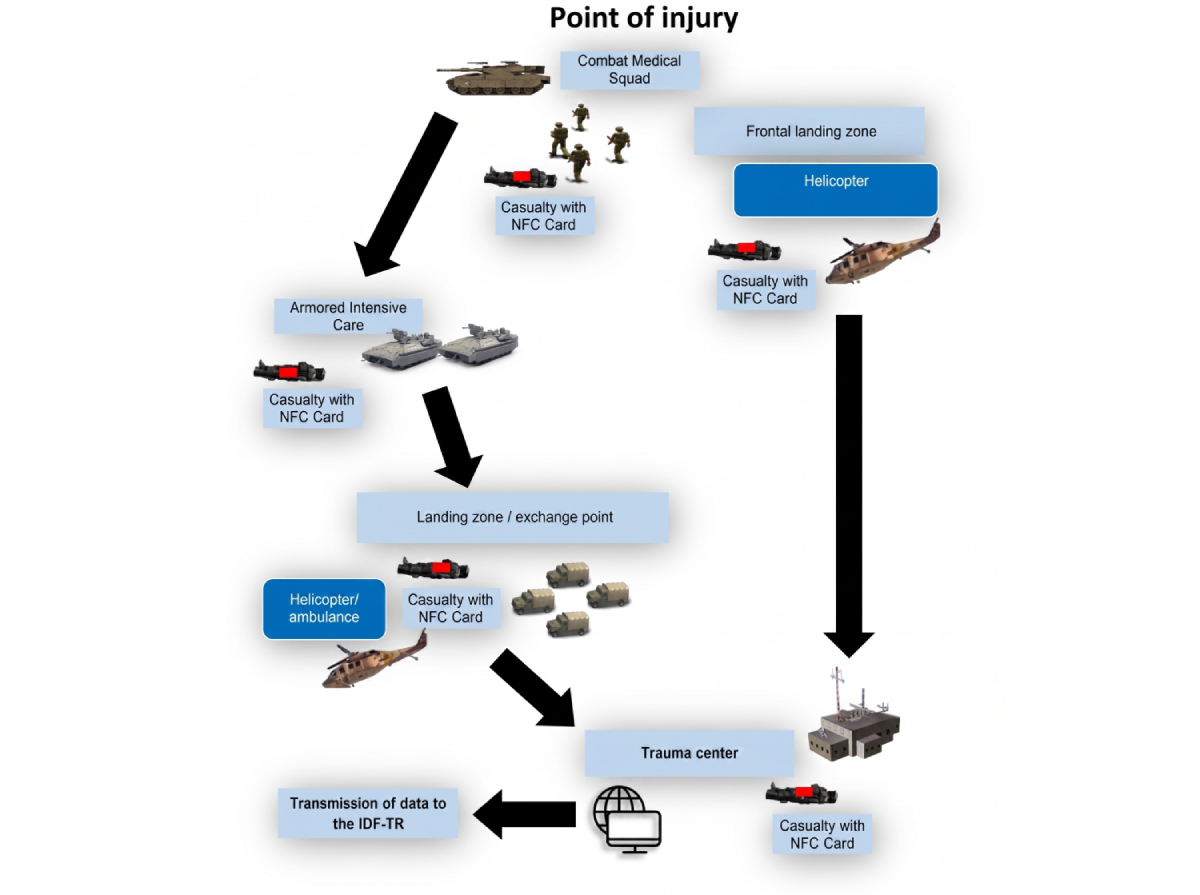
Flow of casualty management and data transfer using the Digital Casualty Card System (DCCS). This figure illustrates the process of casualty management and data transfer from the point of injury to definitive care using DCCS. Casualties are initially managed at the point of injury by a combat medical squad, where a near field communication (NFC) card is attached to the casualty. This card allows for the recording and updating of critical medical information throughout the evacuation chain. At each echelon of care, the NFC card attached to the casualty ensures that medical data are accurately and continuously transferred. Each medical team along the evacuation route is equipped with a tablet that interfaces with the NFC card, enabling them to access, update, and document the casualty’s medical information in real-time. This process continues until the casualty reaches the trauma center. At the trauma center, the information from the NFC card is integrated into the Israel Defense Forces Trauma Registry (IDF-TR). The data are transferred from the NFC card to the hospital’s electronic system using the tablets, where it is then manually entered into the trauma registry. The arrows in the figure indicate the flow of casualties through the different levels of care, with corresponding updates to the medical data on the NFC card at each stage of the evacuation process.

**Figure 5:**
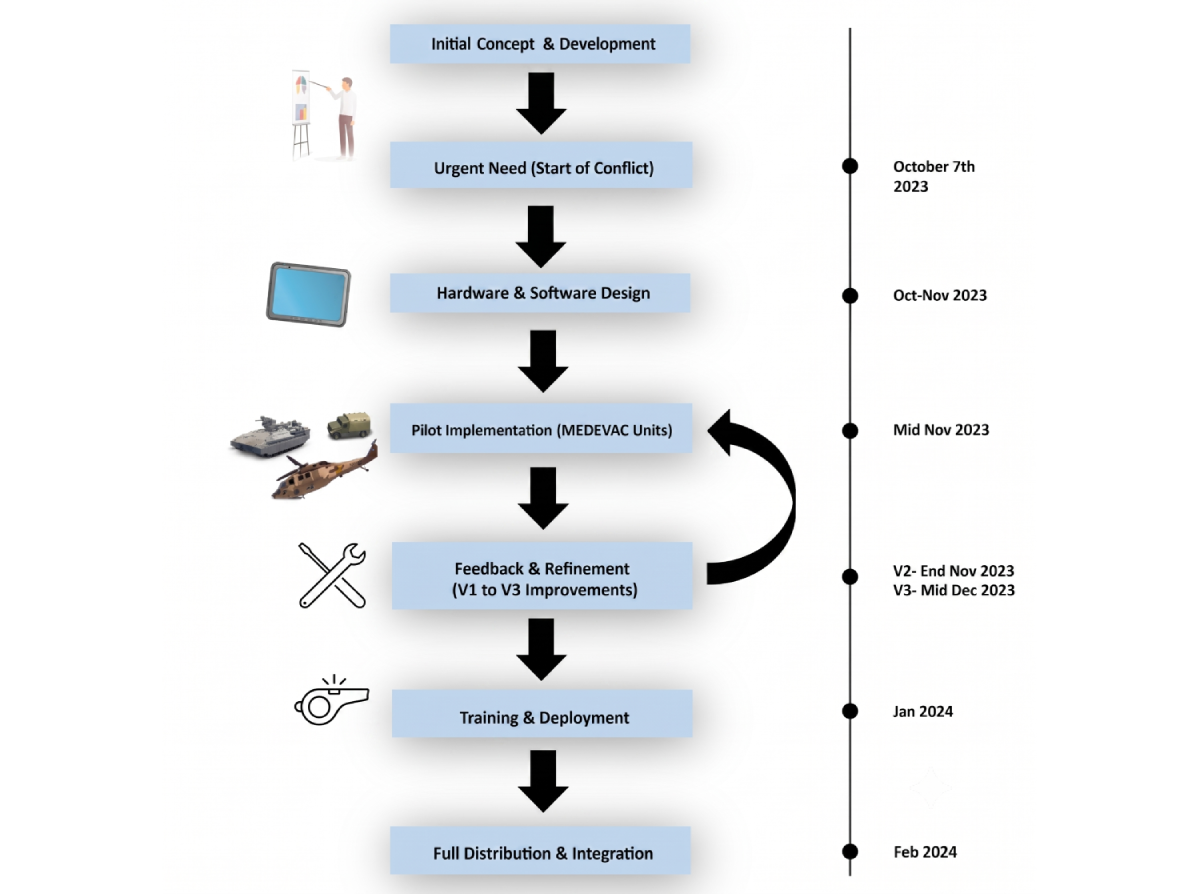
Development and implementation flow of the Digital Casualty Card System (DCCS). This figure illustrates the phased development and implementation process of the DCCS. The process began by the identification of an urgent operational need during the 2023-2024 Israel-Hamas war. A Minimal Viable Product approach was adopted, focusing on a simplified, rapidly deployable solution. The system was first piloted in medical evacuation (MEDEVAC) units before being expanded to broader combat units. Training was conducted through direct instruction and hands-on practice, with iterative feedback leading to refinements across 3 versions (V1 to V3).

Throughout this process, feedback was actively sought from the medical staff across various IDF units. Their suggestions and critiques were invaluable, and they were integrated into subsequent updates to further refine and improve the DCCS. For example, QR code scanning for data transfer between tablets proved too time-consuming and cumbersome under combat conditions, requiring precise alignment and slowing down patient handoff. Version 2 integrated NFC for faster data transfer, but it still required proximity, creating delays and increasing risk under fire. Based on these limitations, version 3 was developed, utilizing an NFC-enabled memory chip embedded in a plastic dog tag, which was placed on casualties upon reaching them. This innovation allowed the data to “move with the casualty,” ensuring access to medical information at all levels of care without requiring device-to-device interaction, significantly improving efficiency and safety in the field. Such continuous feedback loop was therefore crucial in tailoring the system to real operational needs, ensuring its functionality and usability in real-world conditions (Figures 4-5).

**Table 1:** Evolution of Digital Casualty Card System versions and key features.

Version (V)	Key features	Data transfer method	Limitations
V1	Initial pilot version	QR code scanning	Required precise alignment of devices, time-consuming under battlefield conditions
V2	Improved user interface and initial bug fixes	Near field communication (NFC) tags embedded in the tablets.	Required proximity and manual initiation, creating delays and risks under fire or pressure
V3	Bug fixes, interface enhancements, and a Mass Casualty Event management table for improved data tracking and usability	NFC-enabled memory chip in a plastic dog tag attached to the patient accessible by tablets via NFC communication	Some limitations remain, including the continued reliance on manual data entry

### Statistical Analysis and Crosslinking

The data presented are registry-based from the IDF-TR. The IDF-TR is a web-based digital prehospital trauma registry, relying on data from point-of-injury casualty cards (DCCS), after-action reports, and data entry by on scene and en route providers. It contains data collection, including event type, age, sex, mechanism of injury, type of injury, outcome, medical treatment, and type of evacuation.

Categorical variables are presented as frequencies and percentages, with continuous variables as medians and interquartile ranges (IQRs). Statistical analyses were performed using R software (version 4.2.1; R Foundation for Statistical Computing).

## Implementation (Results)

Ultimately, approximately 700 version 3 tablets and 3000 plastic casualty cards were distributed to combat and MEDEVAC medical teams, including ground and aerial evacuation units. Of these, about 500 kits were used operationally and 200 were reserved for training within the IDF-MC and individual units. From the start of the ground operation in Gaza on October 27, 2023, through June 20, 2024, 1175 of 2984 casualties (39%) arrived with DCCS documentation. This reflects gradual implementation, initially targeting key evacuation bottlenecks and later expanding to additional units. During the most intense combat phases, debriefing and data collection were necessarily selective; therefore, the reported figures represent a broad overview, with DCCS coverage and proportions increasing over time. Table 2 summarizes casualty characteristics, Table 3 details mechanisms of injury and treatments, and Table 4 shows system distribution across units and commands.

**Table 2:** Casualty characteristics (N=1175).

Casualty characteristics		Value
Age (y), median (IQR)		22 (20-28)
Male, n (%)		1167 (99.3)
Event type, n (%)		
	Combat	1142 (97.2)
	Routine	33 (2.8)
Population type, n (%)		
	Conscript	569 (48.4)
	Reserve	497 (42.3)
	Military career	109 (9.3)
Casualty urgency, n (%)		
	Nonurgent	472 (40)
	Urgent	676 (58)
	Death	22 (1.9)
	Air evacuation	650 (55)

**Table 3:** Mechanisms of injuries and treatments (N=1175).

			Values, n (%)
Injuries location			
	Upper extremities		432 (37)
	Lower extremities		418 (35)
	Face		320 (27)
	Torso		277 (24)
	Head		141 (12)
	Neck		125 (11)
	Other		92 (7.8)
	Pelvis		87 (7.4)
Body regions involved per casualty			
	1 region		701 (60)
	2 regions		297 (25)
	3 regions		127 (11)
	4 regions		40 (3.4)
	5 regions		9 (0.8)
	6 regions		1 (0.1)
Mechanisms of injury			
	Explosion		710 (60)
	Firearm		179 (15)
	Struck by blunt object		83 (7.1)
	Motor vehicle collision		67 (5.7)
	Piercing object		44 (3.7)
	Fall		37 (3.1)
	Not documented		36 (3.1)
	Other		11 (0.9)
	Burn/electrocution		7 (0.6)
	Poisoning		1 (0.1)
Treatment			
	Hemorrhage control		216 (18.4)
		Tourniquet	175 (15)
		Packing	41 (3.5)
	Volume resuscitation		157 (11.3)
		Whole blood	90 (7.7)
		Freeze-dried plasma	33 (2.8)
		Crystalloids	34 (2.9)
	Definitive airway intervention		22 (1.9)
		Endotracheal intubation	16 (1.4)
		Cricothyroidotomy	6 (0.5)
	Vascular access		
		Intravenous	568 (48)
		Intraosseous	23 (2)

**Table 4:** Systems distributed across units and commands (n=689).

Receiving unit	Systems distributed, n
Medical and evacuation teams in combat units	386
Aeromedical evacuation unit	59
Southern command evacuation teams	20
Northern command evacuation teams	24
Medical training and simulation units	200

## Discussion

### Principal Findings

The rapid development of the DCCS progressing through 3 functional versions within weeks was not merely a technical achievement but a reflection of an exceptional sense of urgency, organizational culture, and leadership support. The outbreak of war created a powerful sense of immediacy that helped to overcome the typical pace of organizational processes. It enabled the team to make fast, pragmatic decisions, accept trade-offs where necessary, and mobilize key personnel, including volunteers and external experts. Crucially, the IDF-MC senior leadership recognized the system's potential early on, granted operational freedom, and actively supported its deployment when needed. The project was led by a dedicated professional team, with high-level organizational support playing a crucial role in aligning resources, removing barriers, and driving the system’s implementation.

Given this urgency, the DCCS was designed as a quick, “low-tech,” and simple solution for easy implementation during an ongoing war. Previous efforts to develop systems incorporating sensors, automatic data capture, and transmission proved infeasible for rapid development and deployment under the given conditions. Consequently, a major limitation of the current system is its reliance on manual data entry—keeping the “man in the loop” introduces the potential for human error and inconsistencies.

Low compliance with prehospital documentation is likely multifactorial. Although a limited awareness about the importance of documentation is a probable contributor, it is not the sole cause. Prehospital medical teams often must perform several tasks simultaneously, sometimes under hostile conditions. These tasks demand significant time and workforce, making documentation one of the first tasks to be neglected, as it may detract from the ability to provide immediate care. However, evidence suggests that mobile apps can significantly enhance documentation practices in deployed environments. Kenney et al [9] demonstrated that user-friendly mobile apps have the potential to improve the completeness, accuracy, and speed of casualty care documentation, even with minimal additional training.

Despite the introduction of DCCS, documentation remains a manual task, now digital rather than paper-based; so, the underlying human factors driving low compliance are not yet fully resolved. DCCS is intended to simplify completion of casualty cards and improve the consistency, completeness, and clinical use of data along the evacuation chain. Accurate documentation also has a crucial legal role: it supports accountability; demonstrates adherence to international standards, Israeli law, and the Geneva convention; and provides clear evidence of care, thereby protecting both patients and providers.

Future development should prioritize on reducing manual data entry by integrating real-time data feeds from monitors and devices, allowing clinicians to concentrate on direct care while improving accuracy and consistency. Speech-to-text solutions powered by generative artificial intelligence could further streamline documentation by enabling hands-free, voice-based recording during treatment. This remains technologically challenging, with few reports from prehospital environments and none from combat settings. Recent work on passive-sensor–based automated documentation in simulated combat casualty care nevertheless suggests a path toward eventual speech-to-text integration [10]. Alignment of these tools with IDF-MC protocols could also provide real-time decision support for critical situations, ultimately enhancing documentation efficiency, care quality, and patient outcomes.

Enhancing DCCS interoperability with other medical and logistical systems could further improve continuity and quality of care. In network-enabled environments, integration with hospital electronic health records, telemedicine platforms, and related systems would allow seamless information flow from point of injury to definitive care. Such integration would also give command and control centers real-time visibility of force health and support more accurate tasking of evacuation assets.

Electronic health record connectivity could help hospitals anticipate incoming casualties, prepare staff and resources, reduce turnaround times for teams and ambulances, and potentially enable automatic billing. Longitudinal studies are needed to assess the long-term effects of digital documentation on patient outcomes, training, and operational efficiency. Sustained success will depend on continuous user feedback, iterative refinement based on field experience, and ongoing technological advances that open new applications and opportunities.

### Limitations

The report's limitations include incomplete data capture due to the reliance on manual entry and variability in the completeness of after-action reports. Since the data documented on DCCS for each patient are manually entered into IDF-TR, the presented data do not fully represent all casualties reported via the system at the time of writing, as some records have not yet been entered into the registry. Additionally, the report is limited to the context of a specific conflict and geographic region, which may affect the generalizability of the findings to other military or civilian settings. The rapid implementation and scaling of the system may have led to variations in training and familiarity among users, potentially impacting the consistency and accuracy of the data collected.

### Conclusion

This project serves as proof of concept for a digitized field medical documentation system, streamlining information transfer between levels of care and ensuring data integrity. The deployment of DCCS during an ongoing war demonstrated substantial improvements in both documentation rates and the quality of data collected in the field compared to traditional paper-based methods. Beyond the technological innovation, the project also reflects an organizational and cultural success: it was driven by a clear sense of urgency, decisive leadership, and a structured, focused, and adaptive development process. The key to its success was a willingness to compromise where necessary, seize emerging operational opportunities, and maintain consistent collaboration between headquarters and field units. DCCS stands not only as a technological achievement but as a model of how mission-driven innovation can be embedded even in complex situations under and during pressure.

## Appendix

Multimedia Appendix 1 i-CHECK-DH Checklist.

## Abbreviations

DCCS: Digital Casualty Card System
IDF-MC: Israel Defense Forces Medical Corps
IDF-TR: Israel Defense Forces Trauma Registry
IDF: Israel Defense Forces
MEDEVAC: medical evacuation
NFC: near field communication

## Data Availability

Data generated or analyzed during this study are available from the corresponding author on reasonable request.
