# Supplementary material for: From Paper to Digital Medical Documentation in the Field: The Rapid Development and Deployment of the Digital Casualty Card System During a War
Source: JMIR Med Inform. 2026 Jul 24;14:e79808. doi: 10.2196/79808 (PMC13451869; doi:10.2196/79808)
Supplement: Supplementary file 1 [file medinform_v14i1e79808_app1.pdf]

"From Paper to Digital Medical Documentation in The Field: The Rapid Development and Deployment of the Digital Casualty Card System During a War"

Preliminary Implementation Report (i-CHECK-DH) Checklist

Item	Addressed	Location in manuscript	Comments
1. Title	✓	Page 1	The manuscript is identified as an Implementation Report and describes the implementation of the Digital Casualty Card System (DCCS).
2. Abstract	✓	Abstract (Page 1)	Background, objectives, methods, implementation/results, and conclusions are reported.
3. Context	✓	Pages 1–2 (Introduction)	The implementation context, operational environment, barriers to documentation, and the setting of the 2023–2024 Israel–Hamas war are described.
4. Problem statement	✓	Pages 1–2 (Introduction)	Low rates of battlefield documentation, impaired continuity of care, and the need for improved documentation are clearly described.
5. Similar interventions	✓	Page 2 (Introduction)	Previous digital documentation efforts, including BATDOK, are discussed and contrasted with the DCCS.
6. Aims and objectives	✓	Pages 2–3 (Introduction; Methods – Overview)	Objectives of improving documentation, continuity of care, and implementing the DCCS are clearly

			stated.
7. Blueprint summary	✓	Pages 3–6 (Methods – Overview; General Approach and Usability; Implementation Process)	The implementation strategy, MVP philosophy, agile development, deployment, and iterative refinement are described.
8. Technical design	✓	Pages 3–5 (General Approach and Usability; Hardware; Software)	Hardware, software, NFC communication, air-gapped architecture, workflow, interoperability, and system rationale are described.
9. Target	✓	Pages 5–6 (Implementation Process)	Target users include combat medics, MEDEVAC teams, aeromedical evacuation units, hospitals, and training units.
10. Data	✓	Pages 3–7 (General Approach and Usability; Statistical Analysis and Crosslinking; Data Availability)	Data lifecycle, registry integration, manual upload process, security limitations, and data availability are described.
11. Interoperability	✓	Pages 3–4; 9 (Methods; Discussion)	Current interoperability with the IDF information system, limitations of registry integration, and future EHR interoperability are discussed.
12. Participating entities	✓	Pages 2–6; 11 (Methods; Acknowledgments; Funding)	The roles of the IDF Medical Corps, Trauma and Combat Medicine Branch, K Health volunteers, and collaborators are described.
13. Budget planning	Addressed	Page 11 (Funding)	No dedicated funding was received. Development relied on existing IDF-MC resources together with volunteer collaboration from K Health.

14. Sustainability	✓	Pages 9–10 (Discussion)	Sustainability is addressed through iterative refinement, continued user feedback, interoperability, AI integration, and future technological development.
15. Coverage	✓	Pages 7–8 (Results)	Operational deployment, system distribution, implementation across combat units, and documentation coverage are reported.
16. Outcomes	✓	Pages 7–8 (Results)	Documentation rates, implementation outcomes, casualty characteristics, injury patterns, and treatments are presented.
17. Lessons learned	✓	Pages 8–10 (Discussion)	Organizational lessons, implementation barriers, success factors, technological limitations, and future recommendations are extensively discussed.
18. Unintended consequences (Non-mandatory)	N/A	Pages 10–11 (Limitations)	No major unintended consequences were identified beyond the implementation limitations discussed in the manuscript.
19. Conclusion	✓	Page 11 (Conclusion)	The manuscript summarizes implementation outcomes, organizational impact, and future implications of the DCCS.
20. General (Non-mandatory)	✓	Page 11 (Funding; Data Availability; Conflicts of Interest; Acknowledgments)	Funding, data availability, conflicts of interest, acknowledgments, and use of generative AI for language refinement are

			disclosed.
